# Investigation of sputum volatiles to classify active tuberculosis: A Pilot Study

**DOI:** 10.1016/j.tube.2026.102762

**Published:** 2026-03-31

**Authors:** Grant S. Ochoa, Graham E. Browse, Jane E. Hill

**Affiliations:** a Shirley Diagnostics, Inc., 4000 Mason Rd, Seattle, WA, 98105, United States of America; b Department of Chemical and Biological Engineering, University of British Columbia, 2360 East Mall, Vancouver, BC, V6T 1Z3, Canada

## Abstract

Tuberculosis (TB) remains a major global health challenge due in part to limitations in rapid and affordable diagnostics. Current diagnostic methods are time-intensive and often inaccessible in resource-limited settings, emphasizing the urgent need for rapid, low-cost screening approaches. One promising strategy involves the analysis of volatile molecules associated with TB-infection. In this study (n=100) we identify 14 sputum-derived volatiles and utilize them to construct a machine learning model that classifies samples by TB status with a sensitivity of 90% and a specificity of 86% across cross-validation folds. The resulting profile provides a foundation for further biomarker validation studies with an expanded sample size and the development of non-invasive breath diagnostics.

## Introduction

The bacterium *Mycobacterium tuberculosis*, which causes tuberculosis (TB) is the leading cause of death from single infectious agent worldwide, infecting more than 10 million people annually and causing the death of more than 1 million people annually[1,2]. A significant barrier to controlling TB is limited access to affordable, accurate, and rapid diagnostics, without which, leads to continued illness and continued transmission in the community. Depending on geography and access to resources, a diagnosis might be obtained via sputum smear, nucleic acid amplification, and/or the gold standard, sputum culture[3]. In practice, these diagnostic methods take days or in the case of culture, weeks, to return a diagnostic result and the vast majority of symptomatic patients tested will be negative TB [2,3]. One way to make this diagnostic process more efficient is to pre-screen or triage symptomatic patients. The World Health Organization (WHO) has set guidance metrics on the development of inexpensive, rapid screening tests [4].

One promising avenue to screen for TB is through the monitoring of volatile molecules associated with TB infection [5–8]. Breath and sputa are the most easily accessed lung samples. Since 2007, several groups have explored breath-based volatile organic compounds (VOCs) as potential biomarkers for tuberculosis [5–15]. These studies demonstrate varying levels of breath panel sensitivity (62-91%) and specificity (65-93%). Sputum has also been used as a source of VOCs to diagnose TB. Over the past 16 years, the Anti-Persoonsmijnen Ontmijnende Product Ontwikkeling (APOPO) group have unequivocally demonstrated that trained African giant pouched rats can detect TB-positive sputum samples by scent, with a sample-wise sensitivity and specificity of 86.7% and 88.4% respectively resulting from 53,181 samples [16–18]. Sputa is a more proximal and, potentially, information-rich sample generated by the lung. To date, there remains a critical information gap: no analytical studies have characterized the volatile profile of TB-positive sputa to determine which molecules are predictive of a person’s TB status.

In this pilot-level study, we evaluate the headspace of 100 sputum samples from patients who showed symptoms of TB. Of these, 50 patients had confirmed TB. We report on a preliminary signature for TB from these samples and also discuss the effect of HIV status and sputum smear grade on the sensitivity of the signature.

## Methods

### Patient Sample Information

Sputum samples were obtained from the FIND (Foundation for Innovative New Diagnostics) biobank (Geneva, Switzerland). All samples originated from South Africa. Samples from 100 individuals included 50 with active pulmonary TB and 50 controls with TB symptoms but microbiologically confirmed as *Mtb*- negative. The TB positive (TB+) samples were confirmed with positive smear and culture tests (S+C+). The TB negative (TB−) samples confirmatory tests were both smear and culture negative (S−C−). Furthermore, all samples were tested and their status confirmed by Gene Xpert MTB/RIF assay. A symptom survey was administered to all patients as part of inclusion criteria, regarding symptoms such as persistent cough, expectoration, hemoptysis, chest pain, dyspnea, malaise, fever, sweats, etc. Furthermore, 40 of the 50 TB− (S−C−) group samples had a positive IGRA (Interferon gamma release assay) test result. Where possible, the TB− group patients were reassessed two months later at a follow-up appointment to check for improvement in symptoms. Five of the TB− group patients were determined to likely have subclinical TB at follow-up. All subjects gave their signed, informed consent to participate and all were at least 18 years old. The sputum samples were originally collected across four collections between the years 2017-2021 with the following ethics approval numbers: 192/2012 (for two collections); B18/07/004; B19/04/005.

### Sputum Preparation

Sputum samples, originally stored neat at −80°C, were thawed in a 4°C refrigerator for 18 hours before aliquoting ~0.3-0.4 g of sputum into 20 mL headspace vials. To each vial, enough sodium chloride (Fisher Scientific, Pittsburgh, PA, USA) was added to create a 40% w/w mixture to increase the concentration of compounds in the volatile space[19]. The samples were then placed in the −20°C freezer to await chemical analysis. Salted sputum headspace vials were pulled from the freezer and placed in a 4°C tray cooler to thaw at least 1 hour prior to chemical analysis. Sputum samples were randomized prior to sampling.

### Instrumentation

Prior to sampling, the SPME-Arrow (PDMS/DVB/CAR phase) was conditioned in the Markes Centri 90 (Markes International Ltd., Bridgened, United Kingdom) inlet at 250°C for 5 min. The sputum samples were then sampled with the SPME-Arrow at 50°C for 20 min, which was then desorbed onto the Markes Centri 90 cold trap for 5 min at 250°C. The cold trap was then rapidly heated to 300°C and desorbed for 3 min onto a Pegasus GC-HRT 4D (LECO corporation, St. Joseph, MI, USA) GC×GC-TOFMS with an Agilent 8890 GC equipped with a Markes Centri 90 sampling system and thermal modulator. The first-dimension column was a Rxi-624SilMS 60 m × 250 μm i.d. × 1.4 μm film thickness, and the second-dimension column was a Stabilwax 2 m × 250 μm i.d. × 0.5 μm film thickness, the injection mode post-cold trap desorption was splitless at a constant flow rate of 2.0 mL/min of grade 5.0 helium (99.999%, Linde, Tacoma, WA, USA). The oven temperature program started at 40°C for 0.2 min, ramped to 235 °C at 7°C/min and held for 12 min with the secondary oven and modulator held at a constant +5°C and +15°C offset from the GC oven respectively. The modulation time was 3 s, with a 0.9 s hot pulse time and 0.6 s cool time. The high resolution TOFMS was employed as the detector, with the following parameters: electron impact at 70 eV, acquisition range: 45-505 *m/z*, acquisition rate: 200 spectra/s, with an ion source temperature of 250°C. One milliliter of internal standard TO-14A mix (Restek, Bellefonte, PA, USA) was added to each tube prior to desorption. Sputum samples were run in batches of 15 samples. Each batch included a blank vial at the front and end of the sequence. Each batch also included four replicates of a standard mix for instrument verification, three run at the beginning of the batch and one at the end. The standard vials were prepared by placing two microcapillary tubes filled with diluted Grob standard (Restek, Bellefonte, PA) and diluted C_8_-C_20_ standard into the vial and sealing.

### Data analysis – ChromaTOF Tile

Initial feature selection was performed by tile-based F-ratio analysis with ChromaTOF Tile (LECO, St. Joseph, MI, USA). After importing the acquired sample files to ChromaTOF Tile, the samples were labeled according to their TB status, resulting in two classes for F-ratio analysis with fifty replicates in each class. A tile size of 4 modulations (12 s) on ^1^D and 40 spectra (200 ms) on ^2^D was selected to account for the average peak width along both dimensions as well as accommodating moderate retention time shifting of ~1 modulation on ^1^D. A signal to noise (S/N) threshold of 30 was selected with two samples required to pass the threshold in order to make the hitlist. A minimum noise cutoff of 500 was selected to prevent low quality tiles from passing the S/N threshold. No F-ratio threshold was set. A minimum of 3 masses were required to pass the S/N threshold within a tile for it to make the hitlist.

After the hitlist was generated, analyte identification was performed by comparing tile spectra against the NIST mass spectral library. Hits belonging to column bleed streaking, such as cyclotrisiloxane, were excluded from the hitlist with retention time exclusion zones. Following this process, the final hitlist, containing 935 hits, and the ChromaTOF TILE-computed peak areas for these hits at the *m/z* with the largest area difference between classes was exported to MATLAB R2021a (The Mathworks Inc., Natick, MA, USA) for further multivariate data analysis.

In addition to NIST library matching, the feature’s tentative assignment molar masses were confirmed with positive chemical ionization when present. While structural isomers are still possible this gives increased confidence to the initial assignment. Retention indices calculated based off n-alkane anchor molecules are provided, however library information for the first-dimension column phase (Rxi-624SilMS) is not available for comparison.

### Multivariate Analysis

Data was cleaned prior to multivariate analysis by the following procedure. Features whose frequency of observation (FOO) with at least 80% in either the TB+ or S−C− class were included. Normalization factors were calculated by applying PQN to the internal standard responses and applied to the data, which was then log_10_ transformed. Missing values were imputed with ½ the minimum value of the feature, after which the data was autoscaled. The data was then batch-corrected using the removal of unwanted variance 2 (RUV2) algorithm, removing 2 components of variance to correct for analytical batch drift[20].

A secondary feature selection scheme, employing Boruta and ReliefF, curated a narrower selection of features likely to distinguish between the TB+ and S−C− classes. The features selected by Boruta were combined with the top 30 features scored by the ReliefF algorithm. Lastly, a final feature selection approach utilizing PLS-DA variable importance in projection (VIP) scores and selectivity ratio (S-ratio) was implemented, resulting in a total of 14 features. Feature significance was tested using the non-parametric Mann-Whitney U test with Benjamini-Hochberg correction for multiple comparisons at a significance level of p<0.05. A classification model was built using partial least squares discriminant analysis (PLS-DA) using these 14 features and cross-validated (CV) using a venetian blinds approach, leaving out 10% of the data on each iteration. A total of 3 latent variables (LVs) were retained, which had the lowest cross validation classification error and number of LVs. A strict prediction score threshold of 0.5 was applied, assigning samples with a score above 0.5 as TB+ and below 0.5 as TB−. To confirm the validity and robustness of the selected features, the VIP/S-ratio feature selection approach was performed within a cross-validation approach with 10 splits chosen by a venetian blinds approach, applying Boruta and then ReliefF and S-ratio to each CV fold. For each fold, the performance of the selected features was assessed by training a cross-validated PLS-DA model, which was then used to predict the holdout samples

## Results and Discussion

We received 100 sputum samples from 100 individuals from the FIND. The 50 subjects designated as active TB had both a positive sputum smear and positive culture result (S+C+) (Table 1); 48/50 of the samples had a positive GeneXpert MTB/RIF^®^ result. The 50 control subjects had generic respiratory (TB-like) symptoms but they did not have any positive TB diagnostic result (S−C−). Patient demographic information is provided in Table 1. The average age between cohorts was similar and 55% of patients were HIV positive. The TB+ patient’s sputum smear grades are reported in Table 1.

**Figure 1.**
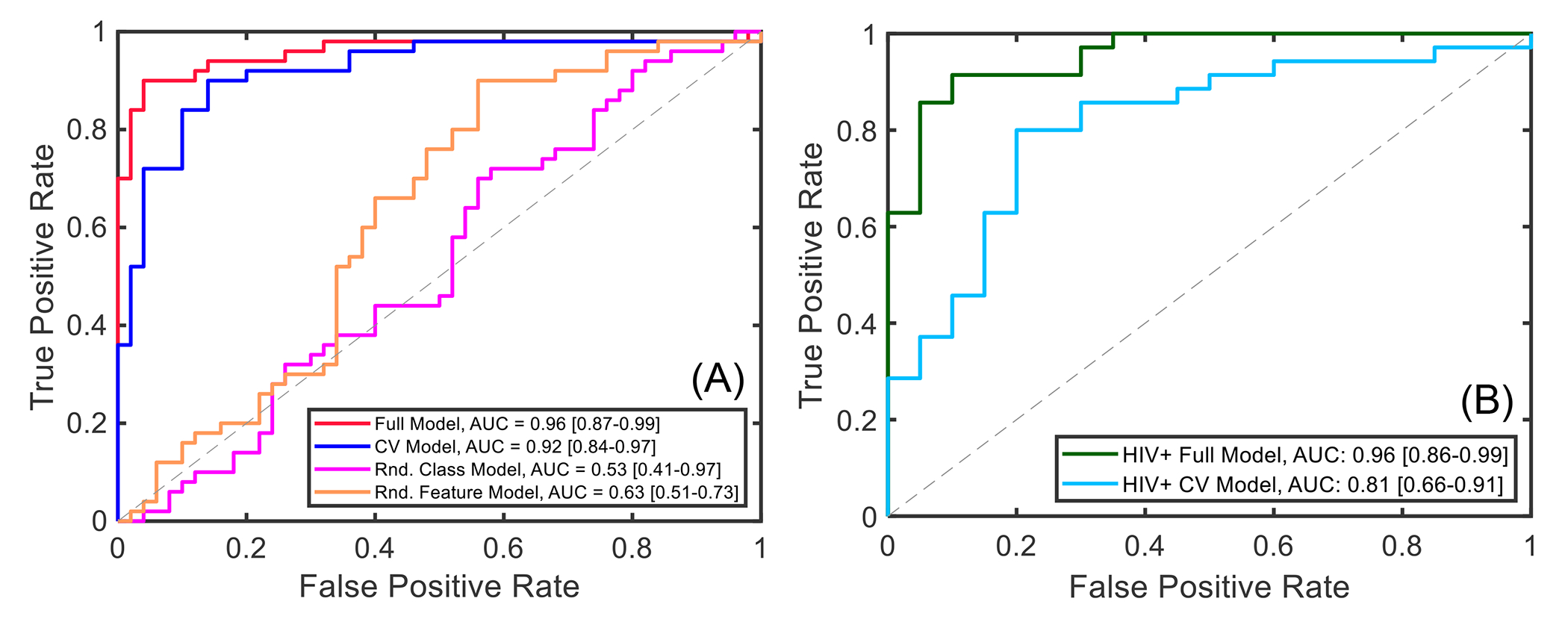
Receiving operator characteristic (ROC) curve for the 14 selected features from the headspace of sputum samples to classify samples as TB− or TB+ (A). The red line represents the full model trained on all samples, while the blue line represents the cross-validated model generated by predicting 10% of the samples and with a model trained on the remaining 90% repeatedly until all samples are predicted. For comparison against random data, the pink line represents the model trained with randomized class labels and the orange line represents the model trained with 14 random volatile features with AUCs of 0.53 and 0.63 respectively. B) The ROC curve for the PLS-DA model trained only on the HIV+ samples to assess potential bias due to class imbalance. The green line is the full model while the cyan line represents the cross-validated (CV) model. Where S+C+ is smear positive culture positive and AUC is area under the curve.

After chemical and chemometric analysis of the sputa headspace, 14 features were extracted as being able to best distinguish between TB+ and TB− samples. The area under the curve (AUC) using these 14 features was 0.96 and the cross-validation model yielded an AUC of 0.92 with a 95% confidence interval (CI) of 0.84-0.97 (Figure 1A), demonstrating that the model was robust even when 10% of samples were excluded from the training step. Furthermore, the AUC remained unchanged when increasing the left-out data percentage to 20% (Figure S1). The sensitivity of the cross validated model with 10% leave-out rate was 0.90 and the specificity was 0.86, meeting the WHO’s triage requirements of ≥0.80 for sensitivity while missing the ≥ 0.98 requirement for specificity when compared to the liquid culture test. To confirm these were not the result of spurious chance, we retrained two random cross-validated models, one with randomized class labels (i.e., the swapping of TB statuses) and the other with 14 random volatile features (Figure 1A). The AUC of these cross validated models were 0.53 and 0.63, respectively. These results suggest that the 14 volatile features are useful for distinguishing TB+ sputa from TB− sputa and deserve further investigation as potential candidates for TB sputum as well as breath biomarkers. The presence of the five TB− group subjects determined to have likely subclinical TB at follow-up could conceivably have a confounding effect on the model performance. A PLS-DA model trained on all samples excluding these 5 subclinical patients resulted with a cross-validated AUC of 0.90, with a similar sensitivity and specificity of 0.90 and 0.82 respectively. Thus, we determined these five likely subclinical TB samples did not greatly affect the model performance. 

World-wide, TB is the most common co-infection for people infected with HIV[21–24]. This attack on the body’s natural defenses not only affects the body’s response to TB infection but potentially how the TB infection progresses, which could affect metabolite expression[25]. To assess if the volatiles emanating from sputum samples from patients who have an HIV co-infection were anomalous from the whole, the classification accuracy for this subgroup was assessed within the full set model – resulting in a classification accuracy of 89%, with a sensitivity of 0.89 and a specificity of 0.85.

A separate model was generated using the volatile molecules exclusively derived from patients with HIV infection with and without TB co-infection. The AUC of the full PLS-DA model of the 55 HIV+ sample subset using 14 volatile features was 0.96 and the cross-validation model AUC was 0.81. The cross-validation model had a reduced sensitivity and specificity of 0.77 and 0.75, respectively, compared to the comprehensive model described above, missing the WHO’s triage requirements of ≥0.80 for sensitivity and ≥0.98 for specificity when compared to liquid culture. However, the overall classification accuracy was 87%, which is similar to the accuracy of HIV+ samples in the comprehensive model. Thus, despite the reduced sensitivity, it is unlikely that the HIV+ samples were significantly different from the HIV− samples.

**Figure 2:**
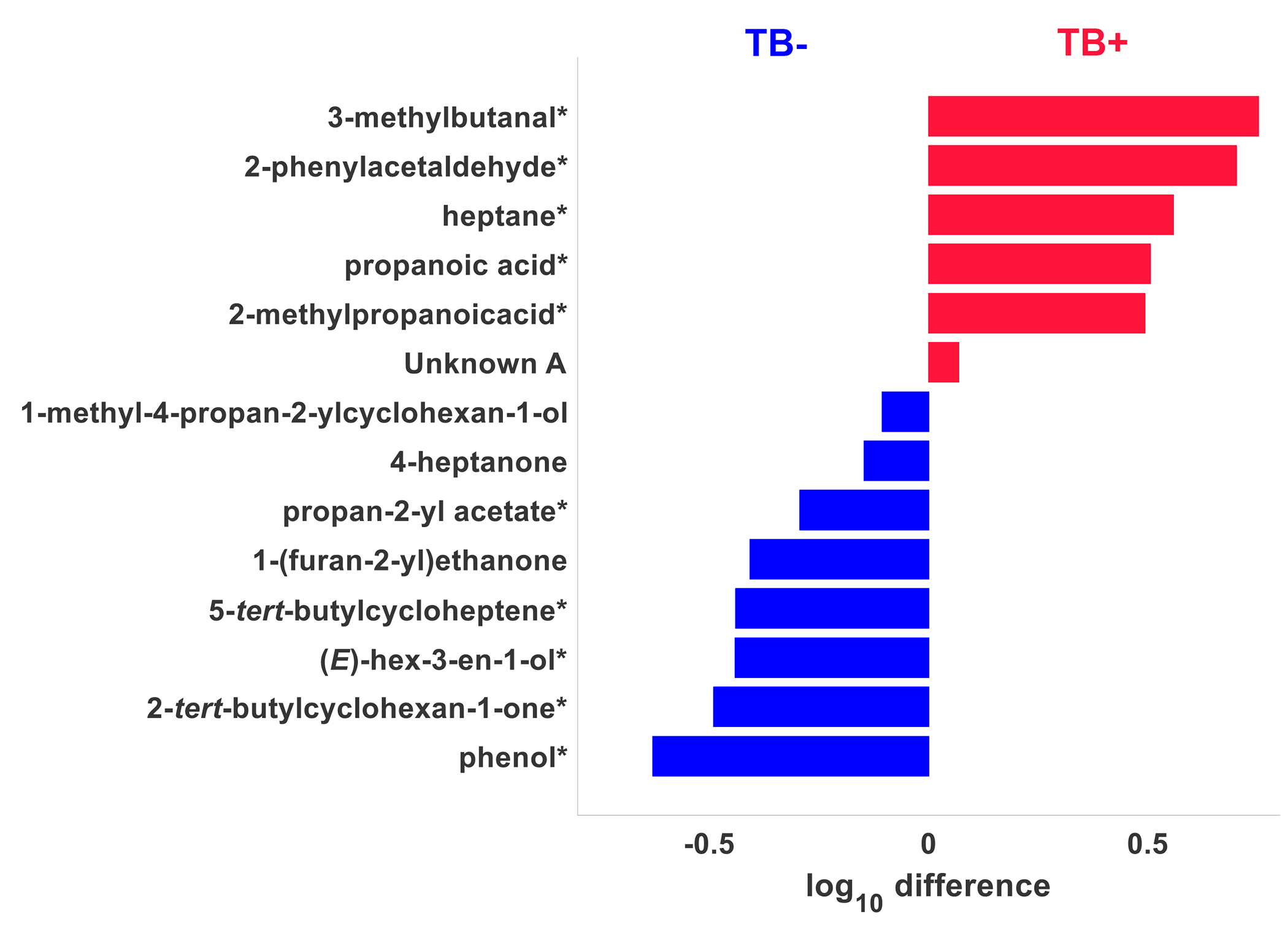
14 selected features and their relative expression in the TB+ and TB− groups of sputum samples. Features with an asterisk (*) were statistically significantly different (p <0.05) between the two groups. The bar length represents the log_10_ difference of the average normalized area for each discriminatory feature.

The set of 14 features from the full model is composed of two alkanes, eleven oxygen-containing molecules, i.e., acids, alcohols and ketones, and one compound did not meet the similarity value required to be tentatively identified. The difference in presence of the 14 headspace features is indicated in Figure 2, with 11 features being statistically significantly different between the TB+ and TB− group. All six features which were expressed predominantly in the TB+ group were present in each of the TB+ samples tested, with the two features with the highest difference in expression both being the aldehydes 3-methyl-butanal and benzeneacetaldehyde. The other chemical classes of the compounds in the TB+ group were an alkane, two acid compounds, and an unknown compound. Of the remaining eight features, three, 4-heptanone, 5-*tert*-butylcycloheptene, and phenol were present in 100% of samples. The remaining 5 features exhibited varying frequencies of observation (FOO) across the TB+ and TB− samples. Specifically, the FOO for 1-methyl-4-propan-2-ylcyclohexan-1-ol was 96% in TB+ samples and 90% in TB− samples. The FOO for propan-2-yl acetate was 82% in TB+ samples and 86% in TB− samples. 1-(furan-2-yl)ethanone was detected in 82% of TB+ samples and 92% of TB− samples. The FOO for (*E*)-hex-3-en-1-ol was 68% for TB+ samples and 86% for TB− samples. Lastly, 2-*tert*- butylcyclohexan-1-one was observed in 90% of TB+ samples and 96% of TB− samples. These FOO are summarized in Table S1 in the supplementary information. Of the selected features, only 3-methylbutanal has previously been reported in *M. Tuberculosis* cultures in vitro and in TB+ breath samples[6,12]. Phenol is a known background contaminant in breath studies utilizing Tedlar bags and is often excluded from analysis, however, since we sampled the sputa directly, it has been included in the final selection[26]. The other 12 selected features have not previously been reported in tuberculosis breath or culture volatile studies.

The stability and robustness of features selected for this model were investigated with a cross-validation feature selection approach. The data was split into ten cross validation folds. Iteratively leaving one fold out, the entire feature selection approach, Boruta and ReliefF followed by VIP/S-ratio, was applied to select features within each CV fold. Across the folds, when accounting for redundant features, the number of features selected ranged from 14-37. In total, there were 51 unique features selected amongst all folds, with 14 features appearing in at least 6 folds. Of these 14 variables, five were shared with the main features of our original model. A table describing the main features appearance rates in the cross-validated folds is available in the supplemental information (Table S2). The range of AUC values of the cross validated models was 0.88-0.94, while the sensitivity ranged from 0.80-0.89 and specificity ranged from 0.73-0.93. This demonstrates that each of these individual models, including the main model in Fig 1, perform similarly well despite having different underlying variable combinations, making each variable set equally valid for initial study. Despite there being a good range of selected variables, the performance of models using these variables all perform similarly well. This underlies the point that the 14 selected main features are not the only variables that produce a good model, but an initial set, chosen conservatively to represent this pilot data. Thus, definitive conclusions as to the finality of the biomarker lists in this study cannot be made and will require follow up study with expanded datasets, as discussed later.

**Figure 3:**
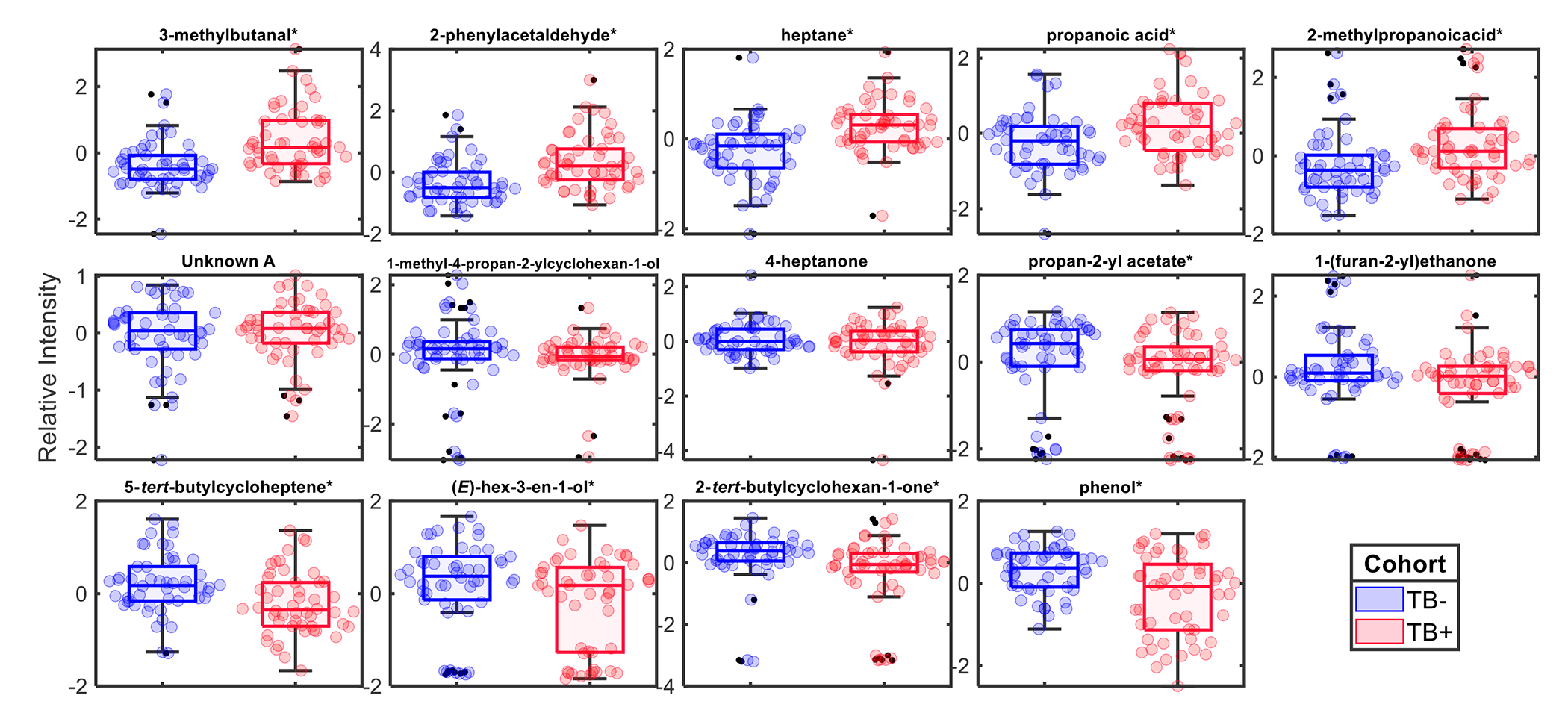
14 selected features and box charts comparing their relative distributions in both the TB− and TB+ classes. The compounds are arranged in order of their log10 difference as in Figure 2. Compounds marked with an asterisk (*) were statistically different between the two groups (p < 0.05).

Figure 3 shows box charts for each of the 14 selected discriminatory features, visualizing the relative distribution of their peak responses between classes. These are sorted in order of their log_10_ difference from Figure 2. While the first 5 features, present with higher means in the TB+ group, are marked as statistically significant, one could not assign a class to a sample based on the response of one of these features alone. The same is true in the opposite sense for the features with higher means in the TB− group, thus, the optimal PLS-DA model utilizes the covariance of these features to assign a classification. If volatile biomarkers from sputum, or breath for that matter, are to be used to develop a diagnostic it will likely be implemented similarly, utilizing a panel of biomarkers, the combination of which will be what determines the classification of a sample.

Sputum smear microscopy serves as the primary method of detection for the 90% of TB patients worldwide who live in low- and middle-income countries [27,28]. Sputum smears are graded by the number of fast acid bacilli present in the smear using a light microscope, rating from scanty to 3+[29]. Patients with low quality sputum smear (scanty, 1+) demonstrate reduced accuracy to tuberculosis diagnostics and recognizing this, the WHO has set sensitivity and specificity targets for patients that are smear-negative that are less stringent compared to smear-positive requirements[30]. Table 1 presents the results of our models cross-validated predication of TB+ samples stratified by their smear grade. The overall accuracy of correctly classifying TB+ samples was 90% while the accuracy for low grade smear (scanty, 1+) was 87.5 % (14/16) and for high grade smear (2+, 3+) the accuracy was 90% (28/31). Three samples had no results for sputum smear and thus could not be assessed. There is no significant difference between the misclassification rate of positive samples and the associated smear grades, however, because of the low sample sizes within each grade, the effect of smear grade on volatile feature response cannot be adequately assessed.

### Study limitations

This pilot study is the first to report the putative identity of sputum headspace biomarkers TB screening, however, the study is limited. The initial sample size is small (100 samples) and most of the samples are from sputum grade 3+. In future studies, we recommend a larger pool of samples in all sputum smear categories as well as suggest an additional category, namely, unconfirmed TB i.e., those who are ultimately classified as likely TB but for whom there is no microbiological confirmation of the bacterium and yet the subject responds positively to anti-TB therapy[31–33]. We would also recommend expanding the geographies and the overall demographics of the patients providing sputum samples, including the capture of smoking, diabetes, diet, and medication use. These study design additions would then allow for both the determination of statistical thresholds of these volatiles and as well as evaluate nuances related to different disease stages or severities as well as potentially identify new biomarkers associated with these levels of severity.

## Supplementary Material

Supplementary Material

## Figures and Tables

**Table 1.** Patient demographic information of FIND sputum samples. Sputum smear grades are given for TB+ patients

	TB+ (n = 50)						TB− (n = 50)
Study Population	N/A	S	1+	2+	3+	Total	
Sputum smear status							
Males	3	1	6	7	9	26	20
Females	0	5	4	2	13	24	30
Age, mean ± SD						38 ± 11	36 ± 10
Male						40 ± 13	37 ± 11
Female						36 ± 9	37 ± 9
HIV Status (Y/N)						35/15	20/30
Male						18/8	5/15
Female						17/7	15/15
Male/Female ratio						26/24	20/30

TB+ is tuberculosis positive, TB− is tuberculosis negative, N/A are samples without smear result, S is scanty smear grade, SD is standard deviation, M and F are male and female, respectively, Y/N is yes and no, respectively.
