# Supplementary material for: Investigation of sputum volatiles to classify active tuberculosis: A Pilot Study
Source: Tuberculosis (Edinb). Author manuscript; Available in PMC 2026 Jul 1. (PMC13322640; doi:10.1016/j.tube.2026.102762)

**Supplementary Material**

**Investigation of sputum volatiles for classification of *M. tuberculosis* infection by multidimensional gas chromatography – high resolution mass spectrometry**

Grant S. Ochoa^†^, Graham E. Browse^†^, Jane E. Hill^†,‡,^*

^†^Shirley Diagnostics, Inc. 4000 Mason Rd, Seattle, WA, 98105, USA

^‡^School of Biomedical Engineering, University of British Columbia, Vancouver, BC, Canada

*Corresponding author: School of Biomedical Engineering, University of British Columbia, Vancouver, BC, Canada

Email address: jane.hill@ubc.ca

**Contents**

[Figure S1. ROC curves for 20% cross validation 2](#_Toc216346730)

[Table S1. Selected features frequency of observation 3](#_Toc216346731)

[Table S2. Main feature selection frequency in cross validation strategy. 4](#_Toc216346732)

[Figure S2: ROC curves for cross-validation feature selection. 5](#_Toc216346733)

Figure S1. ROC curves for 20% cross validation. Receiving operator characteristic (ROC) curve for the partial least squares-discriminant analysis (PLS-DA) model trained on the 14 selected features from the headspace of sputum samples to classify samples as TB- or TB+. All models were cross-validated (CV) using a venetian blinds approach, leaving out 20% of the data on each iteration. (A) The red line represents the full model trained on all samples, while the blue line represents the cross-validated model. (B) The ROC curve for the for the PLS-DA model trained only on the HIV+ samples to assess bias due to class imbalance. The green line is the full model while the cyan line is the cross-validated model. The AUC and accompanying confidence intervals for each model are provided.


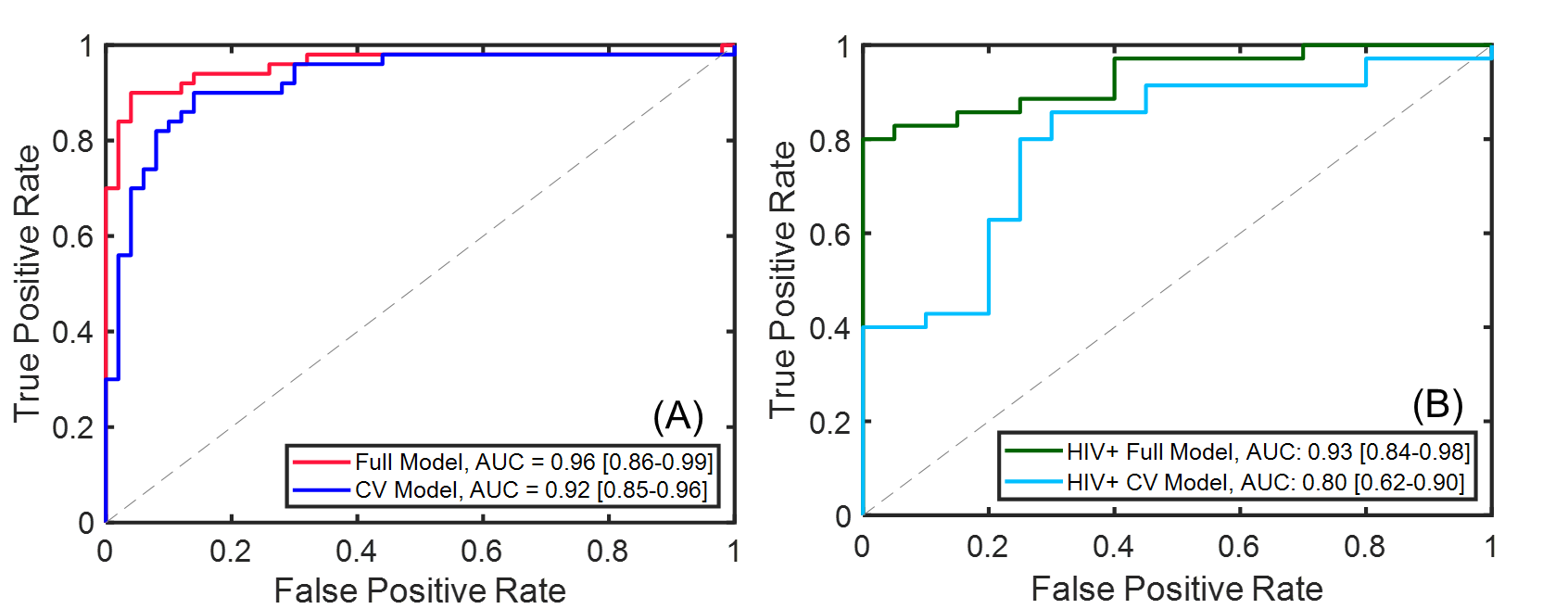


Table S1. Selected features frequency of observation**.** Tabel listing the frequency of observation for each of the selected features in both of the subject categories.

~~
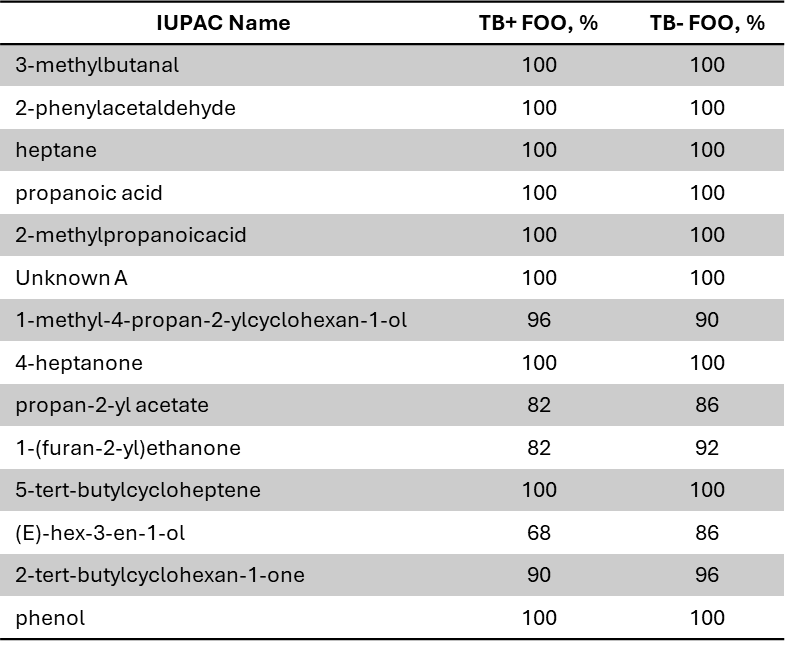
~~

Table S2. Main feature selection frequency in cross validation strategy. Table listing the number of folds each feature from the main selection was chosen in the cross validation strategy for checking the stability of features. A total of 10 folds were tested.


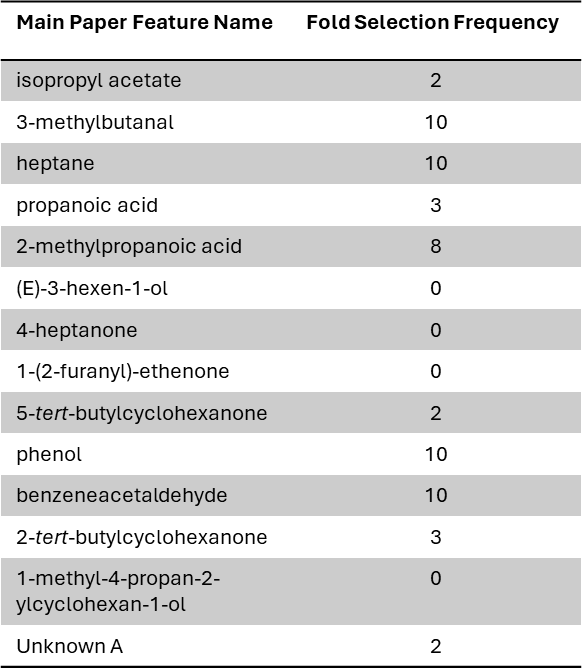


Figure S2: ROC curves for cross-validation feature selection. (A) ROC curves of a model trained on all samples using features which appeared at least 6 out of 10 folds in the cross-validation feature selection approach (Table S2). The cross-validated model, trained with a venetian blinds approach with 10 splits, has an AUC of 0.89 with a 95% confidence interval (CI) of 0.81-0.95). (B) ROC curves of a training/validation split model. Data were randomly assigned to a training and validation set at a split of 75/25. A cross validated model was built with the training set data and the aforementioned features and then applied to the validation set. The cross-validated model has an AUC of 0.86 with a 95% CI of 0.74-0.93. The validation set has an AUC of 0.94 with a 95% CI of 0.77-1.00.


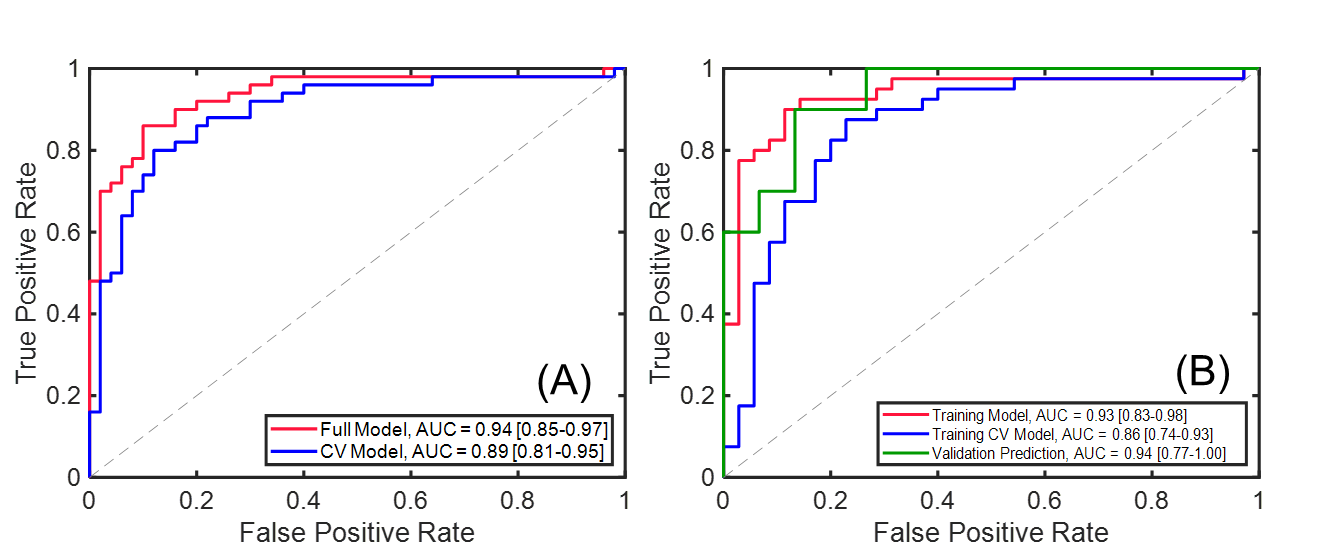

Supplement: Supplementary Material [file NIHMS2167761-supplement-Supplementary_Material.docx]
